# Leveraging Vision Transformers for High-Precision Classification of Cancer Cell Cultures: A Comparative Study on MDA-MB-231 and PC3 Datasets

**DOI:** 10.1109/OJEMB.2026.3666980

**Published:** 2026-02-23

**Authors:** Noreen Fayyaz Khan, Lu Liu, Lucas Bierscheid, John C. Wilkinson, Changhui Yan

**Affiliations:** ^1^ Department of Computer ScienceNorth Dakota State University 3323 Fargo ND 58105 USA; Department of Chemistry and BiochemistryNorth Dakota State University 3323 Fargo ND 58105 USA; Biodesign Center for Personalized Diagnostics, Arizona State University Tempe AZ 85281 USA; ^3^ Department of Chemistry and BiochemistryNorth Dakota State University 3323 Fargo ND 58105 USA; ^4^ Department of Computer Science and the Genomics, Phenomics, and Bioinformatics ProgramNorth Dakota State University 3323 Fargo ND 58105 USA

**Keywords:** Cancer cell classification, CNN variants and ViT, MDA-MB-231 cell line, microscopy image processing, PC3 cell line

## Abstract

*Goal:* Accurate classification of cancer cell cultures is critical for understanding tumor behavior, drug responses, and disease progression. Traditional manual evaluation methods are often subjective and prone to errors, necessitating automated approaches based on deep learning. *Methods:* We design a pipeline using Otsu thresholding, morphological filtering, and watershed segmentation, coupled with class-balanced augmentation. A baseline CNN, two attention-augmented variants: CNN-SE (squeeze-and-excitation) and CNN-CBAM (channel-spatial attention) and a ViT are benchmarked. All models are tuned through a 5-fold cross-validation grid search and evaluated on MDA-MB-231 (triple negative breast cancer) and PC3 (prostate cancer) cell images. The study employed both quantitative and qualitative approaches to comprehensively assess the effectiveness and reliability of the proposed model. *Results:* Attention modules substantially strengthen CNN performance but ViT achieves the best overall accuracy and generalization, particularly reflecting an advantage in modeling long-range dependencies. *Conclusions:* Channel and spatial attention narrows the gap between CNNs and transformers, while transformers provide the highest end-to-end performance. These results support attention-augmented CNNs and ViTs as robust complementary tools for automated analysis of cancer cell cultures.

## I. Introduction

The rapid progress of computer vision has transformed biomedical research, particularly in cancer studies, where accurate classification of cell culture images is critical to understanding tumor behavior, drug response, and disease progression. Manual analysis is error-prone, which highlights the need for automated deep learning approaches. Convolutional Neural Networks (CNNs) remain fundamental in medical imaging due to their hierarchical feature extraction [1]. Their effectiveness has been demonstrated in histopathological analysis, with U-Net becoming a benchmark for biomedical segmentation [2], cell segmentation [3], [4], [5], [6], CNNs that achieve high accuracy in tasks such as large-scale classification and bright field image analysis [7], [8], and actin network classification across cell lines [9], [10], [11], [12], [13]. However, CNNs have limited ability to capture long-range dependencies in biological datasets.

Vision Transformers (ViTs) overcome this limitation by modeling global relationships through self-attention [14], [15], [16]. Unlike CNNs, which rely on local receptive fields, ViTs analyze entire image patches concurrently, enabling the detection of complex patterns and features within images [17]. ViTs have been applied to histopathology, segmentation, and classification [18], [19], [20], detection of retinal diseases [21], and multimodal image fusion [22]. Specialized transformer-based models such as DS-TransUNet and TSE DeepLab [23], [24] further demonstrate their effectiveness. Hybrid models like FTransCNN [25], [26], [27] integrate CNNs and transformers for improved feature extraction and disease diagnosis.

This study systematically compares CNN, CNN-SE, CNN-CBAM [28], [29], [30], [31], and ViTs on Phase-Contrast Microscopy (PCM) images of MDA-MB-231 and PC3 cancer cell cultures. While CNNs have shown success in PCM analysis [32], ViTs’ ability to capture global dependencies [33] leads to superior accuracy and generalization. The contributions of this study are as follows.

• *Comparative analysis:* We systematically evaluated baseline CNN, CNN-SE, and CNN-CBAM architectures against a Vision Transformer (ViT) on PCM images. Attention modules markedly strengthen CNNs (CBAM $>$ SE $>$ baseline), while ViT achieves the best overall performance.
• *Dataset contribution:* This study presents a new dataset of PCM images for the cancer cell lines MDA-MB-231 and PC3, which contributes to new resources for cancer research and imaging analysis.
• *Automated preprocessing framework:* We implemented an automated preprocessing framework that integrates advanced techniques such as Otsu thresholding, morphological operations, and the watershed algorithm to improve the accuracy of cell segmentation. Additionally, data augmentation methods (e.g., flipping, rotation, and Gaussian blurring) are applied to mitigate data sparsity and imbalance.
• *Rigorous evaluation:* We fine-tuned the hyperparameters of CNN and ViT using a grid search based on cross-validation to enhance performance. The models were systematically evaluated using multiple metrics, including accuracy, precision, recall, F1 score, confusion matrices, ROC / ROC-AUC (micro / macro / weighted) and Matthews correlation coefficient (MCC).

## II. Materials and Methods

**Fig. 1.**
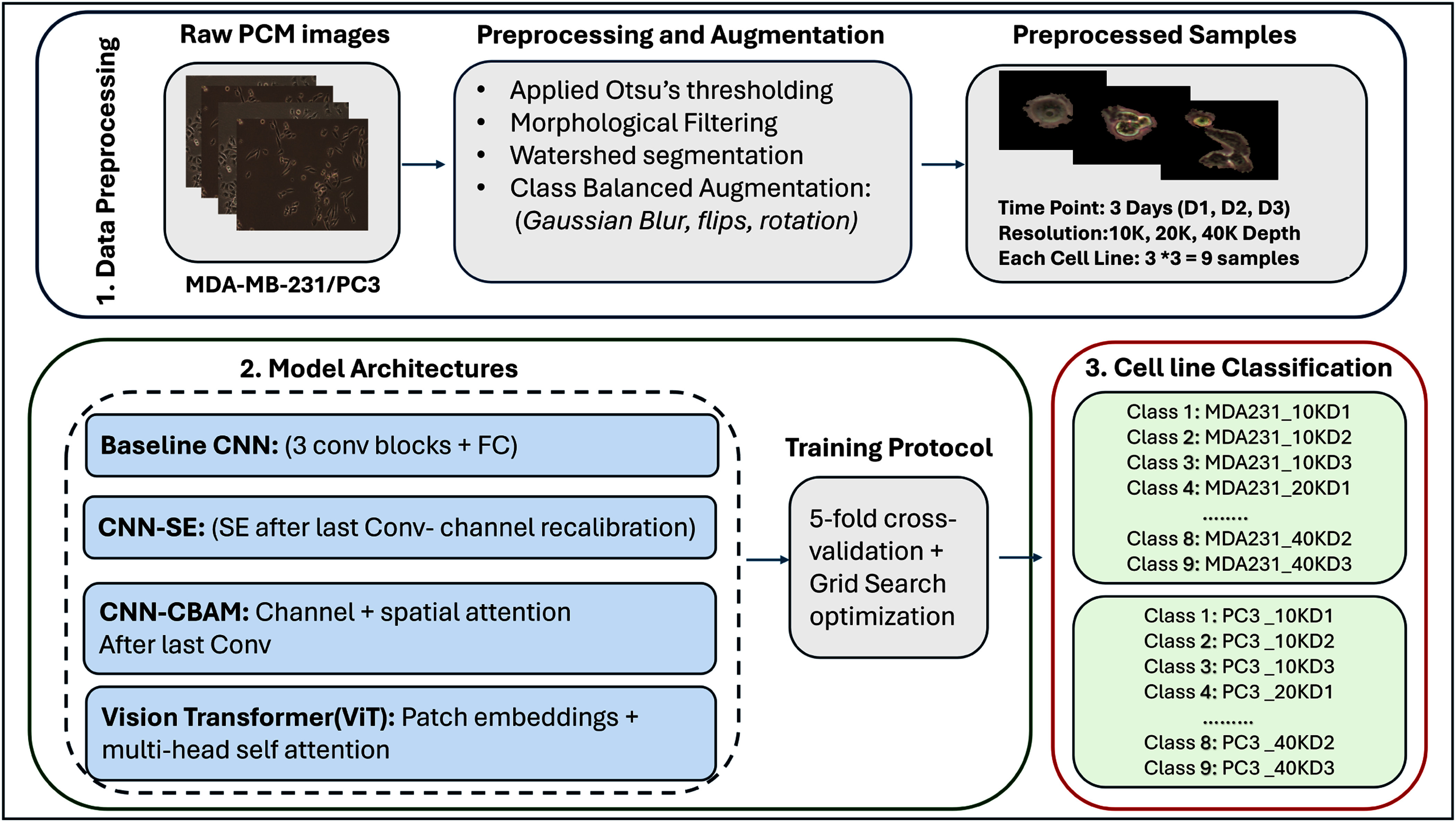
Workflow for PCM cell line classification with attention-enhanced CNNs and vision transformers.

The framework of the proposed system is depicted in Fig. 1. It summarizes preprocessing CNN/SE/CBAM and ViT models, training protocols, and classification of cell lines.

### A. Datasets

#### 1) Data Collection

Microscopy images of two cancer cell lines, MDA-MB-231 and PC3, were obtained using Phase-Contrast Microscopy (PCM). The MDA-MB-231 cell line is a triple negative breast cancer cell culture (TNBC), while PC3 is derived from prostate cancer. Exponentially growing cells were harvested by trypsinization and plated at three different densities: 10,000 cells/well, 20,000 cells/well and 40,000 cells/well. The cells were then cultured in RPMI-1640 + 10 % Fetal bovine serum for three days, during which the cancer cells exhibit different morphological characteristics, e.g. shape and size, depending on cell type, growth condition, and growth time. Cell images were collected each day using a Nikon TS100 microscope with a 10X phase-contrast objective. The images of each cell line were classified into nine classes, which were defined by the combination of initial cell density and growth time. For example, PC3-20KD2 are the class of images of PC3 cells taken at the end of the second day with initial cell density of 20,000 cells/well. There were 6 replicates for each cell line. For each class, 90 images were taken.

#### 2) Data Preprocessing

In order to make the data clean, consistent, and suitable for training deep learning models, we preprocessed the images using a protocol that was specifically tailored to address the challenges of cell culture images. The preprocessing protocol includes two steps, which are image cropping and augmentation. The purpose of image cropping is to obtain images of single cells isolated from their neighbors and the environment. This step is necessary for detecting morphological features of the cells, such as size and shape. The augmentation step extends the diversity of the dataset.

The creation of image files from cell culture to obtain images of individual cells presents unique challenges [34], [35], [36]. Simply cutting the original images into smaller patches, e.g. 500 × 500 pixels, would result in empty patches with no cells for images taken in day 1, and patches with many partial cells in images taken in day 3. A commonly used approach is to isolate single cells using segmentation and patch each cell image to a preferred size with a black background. This normalization improved consistency in cell size and structure, aiding model interpretability. Although dense clusters in Day 3 images posed segmentation challenges due to overlapping cells, the black background helped simplify visual input. Despite concerns that removing the natural context might affect classification, our results show that this method yielded high precision, supporting its effectiveness in this setting. This contrasts with earlier findings that emphasized the importance of contextual information for dense cell classification [2].

Algorithm 1: **Cell Isolation from Raw Microscopy Images.**
**Input:** A set of raw microscopy images $I_{1}, I_{2},\ldots, I_{m}$
**Output:** A dataset of isolated cell images (cropped to a maximum of $500 \times 500$ pixels, excluding marginal or very small cells)
1: **for** each image $I$ in the dataset **do**
2:
$gray \gets \mathrm{convertToGray}(I)$

3:
$binary \gets \mathrm{OtsuThreshold}(gray)$

4:
$binary \gets \mathrm{morphologyOps}(binary)$

5:
$binary \gets \mathrm{fillGaps}(binary)$

6:
$markers \gets \mathrm{watershedSegmentation}(I, binary)$

7:
$boundingBoxes \gets \mathrm{detectCellRegions}(markers)$

8: **for** each bounding box $b$ in $boundingBoxes$ **do**
9: **if** $\mathrm{isValidCell}(b)$ **then**
10:
$cell \gets \mathrm{crop}(I, b)$

11:
$cell \gets \mathrm{limitSize}(cell, 500, 500)$

12:
$\mathrm{save}(cell)$

13: **end if**
14: **end for**
15: **end for**

**Fig. 2.**
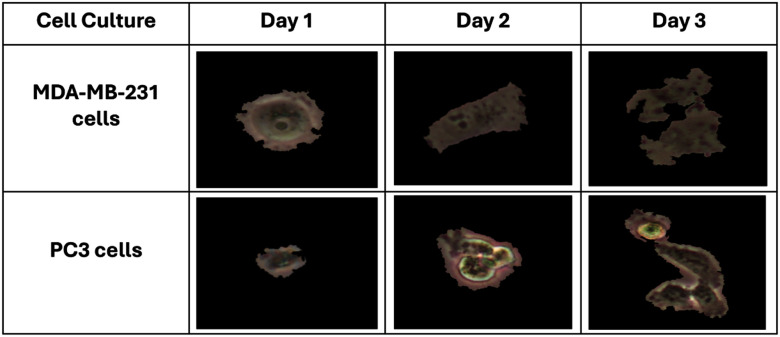
Isolated samples of two Cell Culture with black background: PC3 cells, MDA-MB-231 cells.

To overcome these challenges, we implemented a method to isolate and normalize single cell images following the strategy proposed by Hofener et al. [37]. The details of the implementation are illustrated in Algorithm 1. The segmentation began by converting microscopy images to grayscale and applying Otsu’s thresholding to generate binary masks. Morphological filtering with a 3 × 3 kernel was then used to remove noise and bridge small gaps between cells. Watershed segmentation was applied to separate touching or overlapping cells, ensuring that each cell region was uniquely labeled. Individual cells were extracted by identifying contours and cropping bounding boxes around each cell, while objects smaller than 1000 pixels or larger than 500 × 500 pixels were excluded. Each extracted image was standardized to 500 × 500 pixels for consistency. To assess segmentation quality, we included qualitative examples with binary mask overlays (see Algorithm 1 and Fig. 1). In addition, few random manually annotated images from MDA-MB-231 and PC3 cultures were used to calculate the Intersection-over-Union and Dice coefficients, achieving IoU = 0.91 $\pm$ 0.04 and Dice = 0.94 $\pm$ 0.03, confirming accurate single-cell extraction with minimal boundary loss. Representative examples of the process are shown in Fig. 2. For deep models training, these cropped cells were then resized to 224 × 224 pixels, which is the standard CNNs/ViT input dimension.

Image augmentation is a commonly used approach to address data sparsity and imbalance. In this study, we used an augmentation step that involved a series of transformations, including horizontal flip, rotations within -45$^{\circ }$ to 45$^{\circ }$, and Gaussian blurring. The augmentation method was applied to isolated cell images until each class contained 1,000 isolated cell images. The augmented dataset was then divided into training, validation, and testing subsets in a stratified manner to maintain class balance across all sets.

This preprocessing pipeline resulted in a robust dataset for downstream steps of model training, ensuring that the data were clean, consistent, diverse and balanced. The resultant dataset consists of 9 classes for each cell line, each class having 1,000 images, as shown in Table I.

**TABLE I:** PC3 and MDA-MB-231 Dataset Split (Each Class Contains 1000 Images)

**No.**	**PC3 Classes**	**Images**	**MDB-231 Classes**	**Images**
1	PC3_10kD1	1000	MDA231_10kD1	1000
2	PC3_10kD2	1000	MDA231_10kD2	1000
3	PC3_10kD3	1000	MDA231_10kD3	1000
4	PC3_20kD1	1000	MDA231_20kD1	1000
5	PC3_20kD2	1000	MDA231_20kD2	1000
6	PC3_20kD3	1000	MDA231_20kD3	1000
7	PC3_40kD1	1000	MDA231_40kD1	1000
8	PC3_40kD2	1000	MDA231_40kD2	1000
9	PC3_40kD3	1000	MDA231_40kD3	1000

### B. Classification

This study evaluates and compares deep learning models, baseline Convolutional Neural Networks (CNN), CNN-SE, CNN-CBAM,and Vision Transformer (ViT) for classifying cancer cell images of MDA-MB-231 and PC3. To ensure methodological fairness, all models, including the Vision Transformer, were trained entirely from scratch using the proposed dataset. No pre-trained weights or external datasets were utilized during training.

#### 1) Convolutional Neural Networks (CNN, CNN-SE, CNN-CBAM)

The baseline CNN employs three convolutional blocks (Conv2D-ReLU-MaxPool) with 32, 64 and 128 filters of size 3 × 3, batch normalization after each convolution, a fully connected layer of 256 neurons followed by dropout (0.5) and a softmax output layer. While effective, it treats all feature channels equally, limiting its discriminative capacity in biomedical datasets. To address this, two attention-based variants were implemented. The SE and CBAM modules were inserted after the final convolutional block and before the global average pooling layer to emphasize high-level channel (SE) or channel–spatial (CBAM) features prior to classification. CNN-SE integrates Squeeze-and-Excitation blocks, where global average pooling captures contextual information and channel weights emphasize key biological features. This improves the detection of cellular morphologies in MDA-MB-231 and PC3. CNN-CBAM further extends this by combining channel and spatial attention, highlighting important image regions such as nuclei boundaries and morphological variations. This dual-attention strategy improves sensitivity to subtle inter-cell line differences. All CNN variants were trained under identical preprocessing and augmentation pipelines, with hyperparameters optimized through 5-fold cross-validation grid search to ensure fair comparison.

#### 2) Vision Transformer (ViT)

**Fig. 3.**
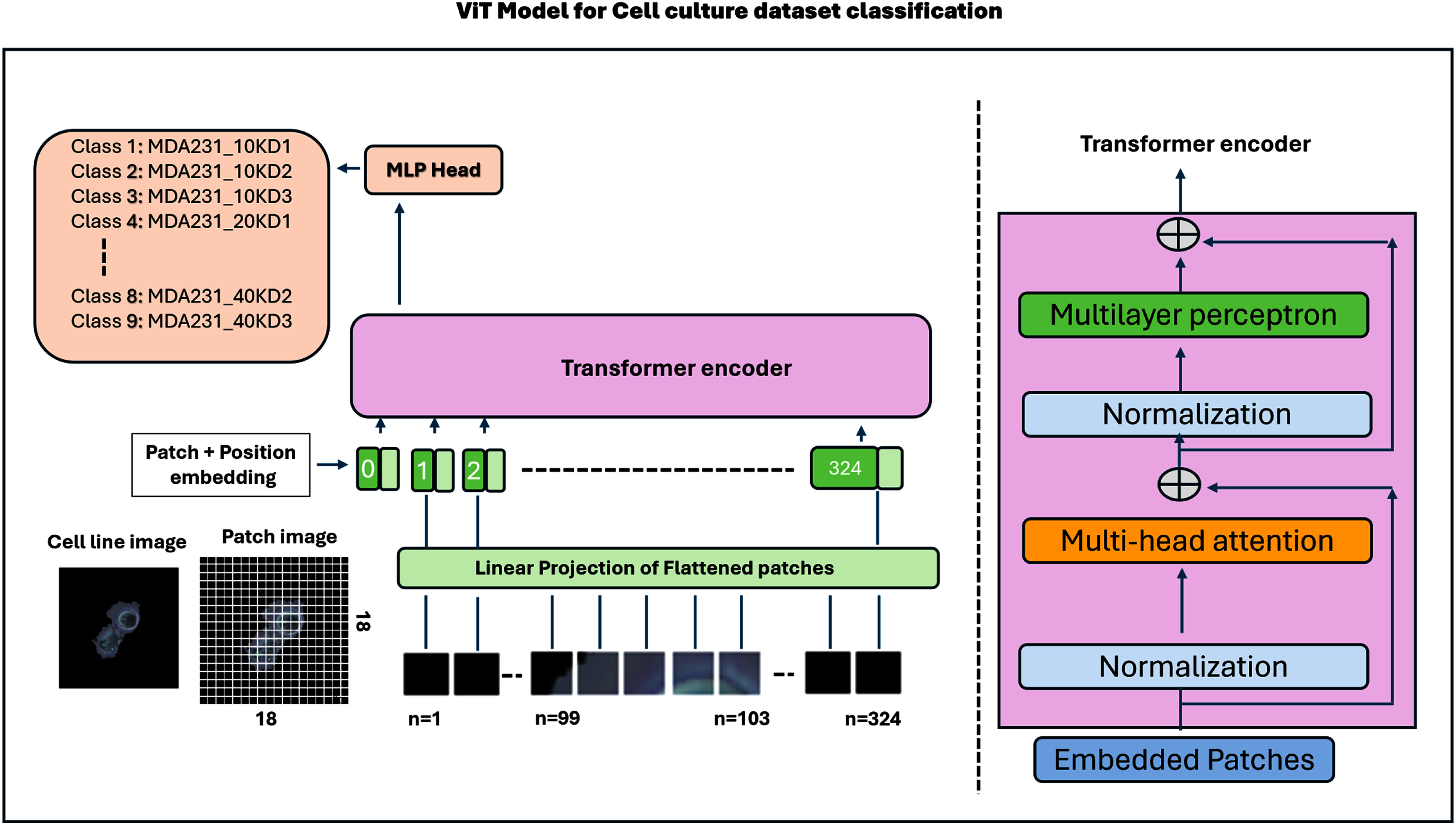
Workflow for vision transformer(ViT)-based image classification with hyper-parameter optimization.

The Vision Transformer (ViT) reformulates image classification as a sequence modeling task by dividing an image into fixed-size patches that are linearly embedded as input tokens, augmented with a learnable class token and positional embeddings to preserve spatial context, and processed through successive Multi-Head Self-Attention (MHSA) and MLP layers, with the final class token representing the classification output [38], [39], [40]. The proposed model is a compact ViT architecture inspired by the ViT-Small family, adapted to biomedical image resolution and dataset scale. The architectural details and parameter configuration of the proposed ViT are listed in Table II. Specifically, the model employs a patch size of 12 × 12 to increase spatial resolution, resulting in 324 image tokens for a 224 × 224 input. Each token is projected to a 64-dimensional embedding, followed by 8 self-attention heads and 8 transformer encoder layers. The feed-forward network uses hidden dimensions of 512, following the standard ViT scaling rule. This configuration is most closely comparable to a ViT-Small/12 variant, but with a reduced embedding dimension and encoder depth to improve computational efficiency and mitigate overfitting on our specialized cell-culture dataset. The total parameter count is approximately 6–7 million, making it significantly lighter than the original ViT-Small ( 22 M parameters), while retaining sufficient representational capacity for fine morphological distinctions in cancer cell images. As illustrated in Fig. 3, each ViT input image is resized to 224 × 224 pixels, and the model divides it into 12 × 12 pixel patches, yielding 18 × 18 = 324 patches per image. Each patch therefore contains 432 elements (12 × 12 × 3 channels). Although 224 is not exactly divisible by 12, the ViT implementation automatically applies zero-padding to align the input dimensions for uniform patch extraction. These embeddings, combined with positional information, were processed by transformer layers to capture long-range dependencies and spatial relationships across the images, enabling effective classification of MDA-MB-231 and PC3 cancer cell cultures. The equation is given by:

\begin{align*}
 z_{0} = \left[ x_{\mathrm{class}};\, x_{1}^{pE};\, x_{2}^{pE};\, \dots;\, x_{N}^{pE} \right] + E_{\mathrm{pos}} \tag{1} 
\end{align*}

where:

• $x_{\mathrm{class}}$: Learnable classification token.
• $x_{i}^{pE}$: Projected patch embeddings.
• $E_{\mathrm{pos}}$: Positional embeddings.

**TABLE II:** Parameter Breakdown of the Proposed Vision Transformer Model

**Parameter**	**Value**	**Typical ViT Reference**	**Comment / Implication**
Image size	224 × 224	Standard across all ViTs	Same as official variants
Patch size	12 × 12	16× 16in ViT-Base/Small	12×12 gives 18×18 = 324 patches; finer spatial resolution
Projection dimension	64	192 (Small) / 384 (Base) / 768 (Large)	Smaller embedding $\rightarrow$ lighter model
Number of attention heads	8	ViT-Small = 3, ViT-Base = 12	Slightly higher head count per embedding dimension
Transformer layers	8	ViT-Small = 12, ViT-Base = 12	Fewer encoder blocks, shallower transformer
MLP head units	[512, 512]	Similar to ViT-Small (2×embed_dim)	Matches small-variant scaling rule
Number of classes	9	Task-specific (e.g., ImageNet = 1000)	For cell-culture dataset
Input shape	(224, 224, 3)	Same	Identical configuration
Parameters (approx.)	$\sim$ 5–7M	ViT-Small $\approx$ 22M, ViT-Base $\approx$ 86M	Compact and computationally efficient
Patch tokens	324 (18×18 grid)	196 (14×14 grid in patch16)	Higher spatial granularity

The tokens are normalized, processed through multi-head self-attention, and then the result is added back to the original tokens. These layers use multi-head attention mechanisms to capture complex relationships in the image data [14]: The equation is given by:

\begin{align*}
 z^{\prime } = \mathrm{MSA}\bigl (\mathrm{LN}(z)\bigr) + z \tag{2} 
\end{align*}

where:

• $\mathrm{LN}(z)$ denotes layer normalization applied to $z$.
• $\mathrm{MSA}$ represents the multi-head self-attention operation.

A feed-forward network that applies a non-linear activation (GELU) and dropout to process information locally. The equation is given by:

\begin{align*}
 z^{\prime \prime } = \mathrm{MLP}\bigl (\mathrm{LN}(z^{\prime })\bigr) + z^{\prime } \tag{3} 
\end{align*}

where:

• $\mathrm{LN}(z^{\prime })$ denotes layer normalization applied to $z^{\prime }$.
• $\mathrm{MLP}$ represents the multilayer perceptron block.

The MLP typically consists of two linear transformations with a GELU activation in between: The equation is given by:

\begin{align*}
 \mathrm{MLP}(x) = W_{2} \, \mathrm{GELU}\bigl (W_{1} x + b_{1}\bigr) + b_{2} \tag{4} 
\end{align*}

where:

• $W_{1}$ and $W_{2}$ are the trainable weight matrices.
• $b_{1}$ and $b_{2}$ are the corresponding bias vectors.
• $\mathrm{GELU}$ denotes the Gaussian Error Linear Unit activation function.

After processing through all Transformer layers, the output corresponding to the class token is normalized to obtain the final image representation for classification. The equation is given by:

\begin{align*}
 y = \mathrm{LN}\left(z_{0}^{L}\right) \tag{5} 
\end{align*}

where:

• $z_{0}^{L}$ is the output corresponding to the class token from the last Transformer encoder layer.

A 5-fold cross-validation strategy was applied on the training set to fine-tune the Vision Transformer (ViT) through a grid-search optimization process. Multiple configurations were systematically evaluated to assess their influence on model accuracy, loss, and generalization. The optimal configuration was obtained with a learning rate of 0.0001, eight transformer layers, eight attention heads, batch size 64, and 200 epochs, achieving the best trade-off between performance and convergence stability. After training, the model was evaluated on the independent test set using accuracy, confusion matrices, classification reports, and ROC curves to validate its discriminative ability across all classes. This process leveraged the ViT’s capability to model complex spatial dependencies for efficient classification of cell-culture images.

## III. Results

Separate models were trained and tested for cell lines MDA-MB-231 and PC3. The trained models were then tested on the test datasets. The results presented in Table III represent aggregate mean values from 5-fold cross-validation, computed across all training folds for each model and dataset. Each fold used 80% of the data for training and 20% for validation, ensuring class balance. The final metrics (precision, recall, F1-score, accuracy, and MCC) reported in Table III, correspond to the average performance across the five folds for both MDA-MB-231 and PC3 datasets.

**TABLE III:** Performance Comparison of CNN, CNN-SE, CNN-CBAM, and VIT on MDA-MB-231 and PC3 Datasets

**Metric**	**Dataset**	**CNN**	**CNN-SE**	**CNN-CBAM**	**ViT**
Precision	MDA-MB-231	0.644	0.69	0.72	0.85
	PC3	0.942	0.95	0.96	0.99
Recall	MDA-MB-231	0.64	0.69	0.70	0.84
	PC3	0.94	0.95	0.96	0.99
F1-score	MDA-MB-231	0.64	0.69	0.70	0.88
	PC3	0.94	0.95	0.96	0.98
Accuracy	MDA-MB-231	0.64	0.69	0.71	0.84
	PC3	0.945	0.95	0.96	0.99
MCC	MDA-MB-231	0.64	0.69	0.71	0.826
	PC3	0.94	0.95	0.96	0.98
Macro Avg	MDA-MB-231	0.66	0.69	0.72	0.88
	PC3	0.93	0.95	0.96	0.99
Weighted Avg	MDA-MB-231	0.66	0.69	0.72	0.88
	PC3	0.94	0.95	0.96	0.99

### A. CNN, CNN-SE, and CNN-CBAM Evaluation on MDA-MB-231 and PC3 Images

The baseline CNN model achieved strong performance on the PC3 dataset (precision, recall, and F1-score all 0.94), but moderate performance on the more challenging MDA-MB-231 dataset (precision, recall, and F1-score around 0.64).

By contrast, CNN-SE demonstrated clear improvements in both datasets. On MDA-MB-231, it raised precision and recall to 0.69, reducing misclassifications across closely related classes. On PC3, CNN-SE achieved near-perfect performance, with precision and recall at 0.95.

CNN-CBAM further improved classification by leveraging both channel and spatial attention. On MDA-MB-231, it reached a precision of 0.72, recall of 0.70, and F1-score of 0.70, indicating better generalization across morphologically variable cell classes. On PC3, CNN-CBAM matched or slightly surpassed CNN-SE, achieving precision and recall at 0.96 with consistent per-class performance.

Receiver Operating Characteristic (ROC) curves [41] of the test experiments are shown in Fig. 5. MDA-MB-231, CNN-SE and CNN-CBAM produced higher AUC values (0.95–0.96) compared to baseline CNN (0.94), reflecting improved discrimination between classes. For PC3, both attention-based CNNs achieved nearly perfect AUC values (0.99–1.00).

**Fig. 4.**
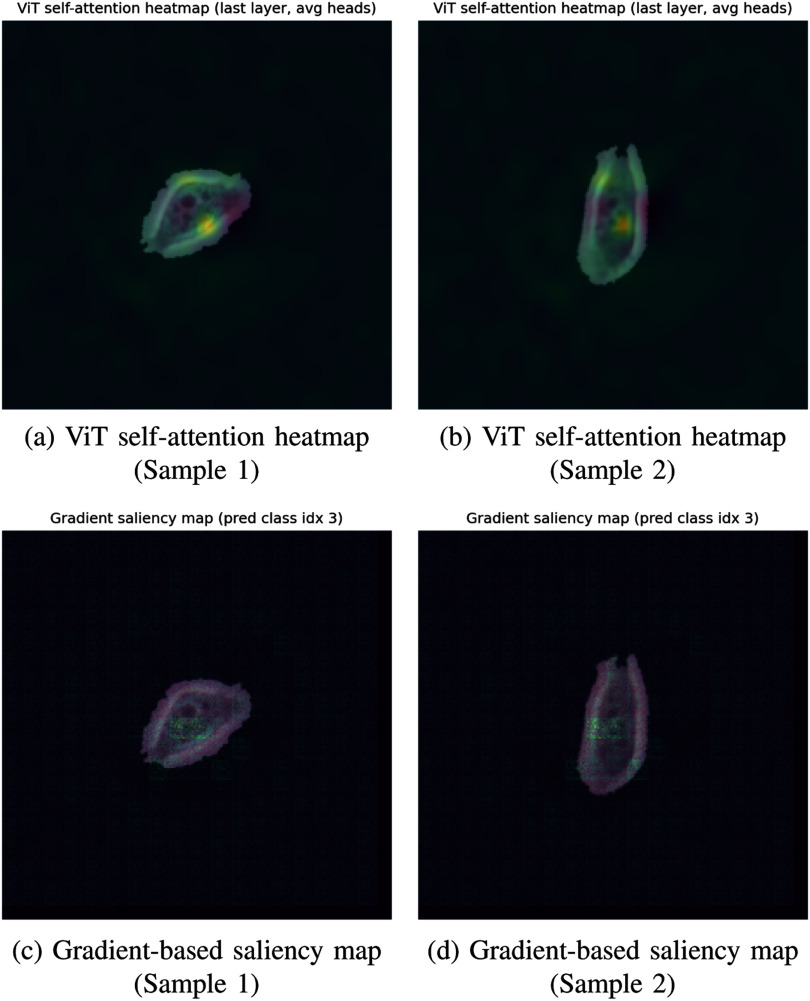
Attention and saliency visualizations of the proposed ViT model, highlighting biologically relevant cell regions that guide interpretable classification decisions.

**Fig. 5.**
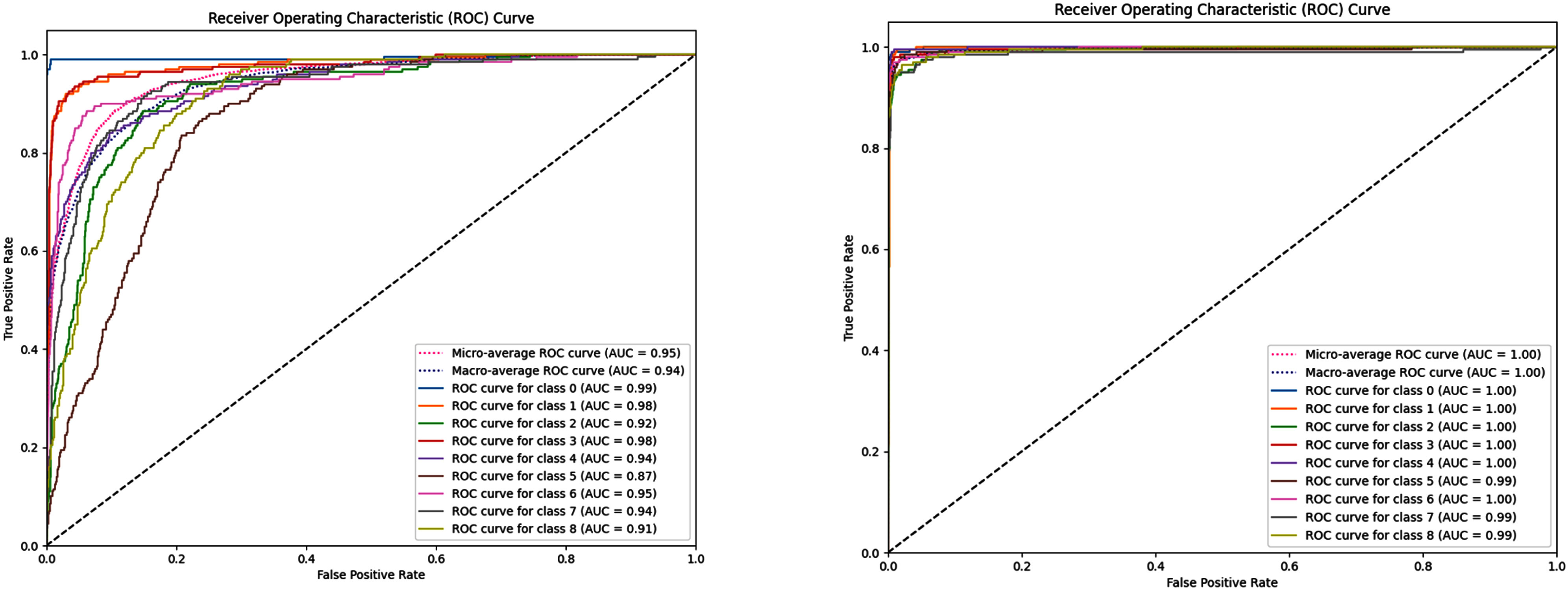
CNN classification performance comparison between MDA-MB-231 and PC3 cell lines using ROC analysis.

These results show that CNN models achieve high classification performance in both datasets, with MDA-MB-231 showing slightly lower performance and higher variation between classes. This may reflect differences in cell line characteristics.

### B. ViT Evaluation on MDA-MB-231 and PC3

Separate ViT models for MDA-MB-231 and PC3 were also trained and tested following the same procedure as CNNs. The models were trained and validated using cross validation on the training set.

**Fig. 6.**
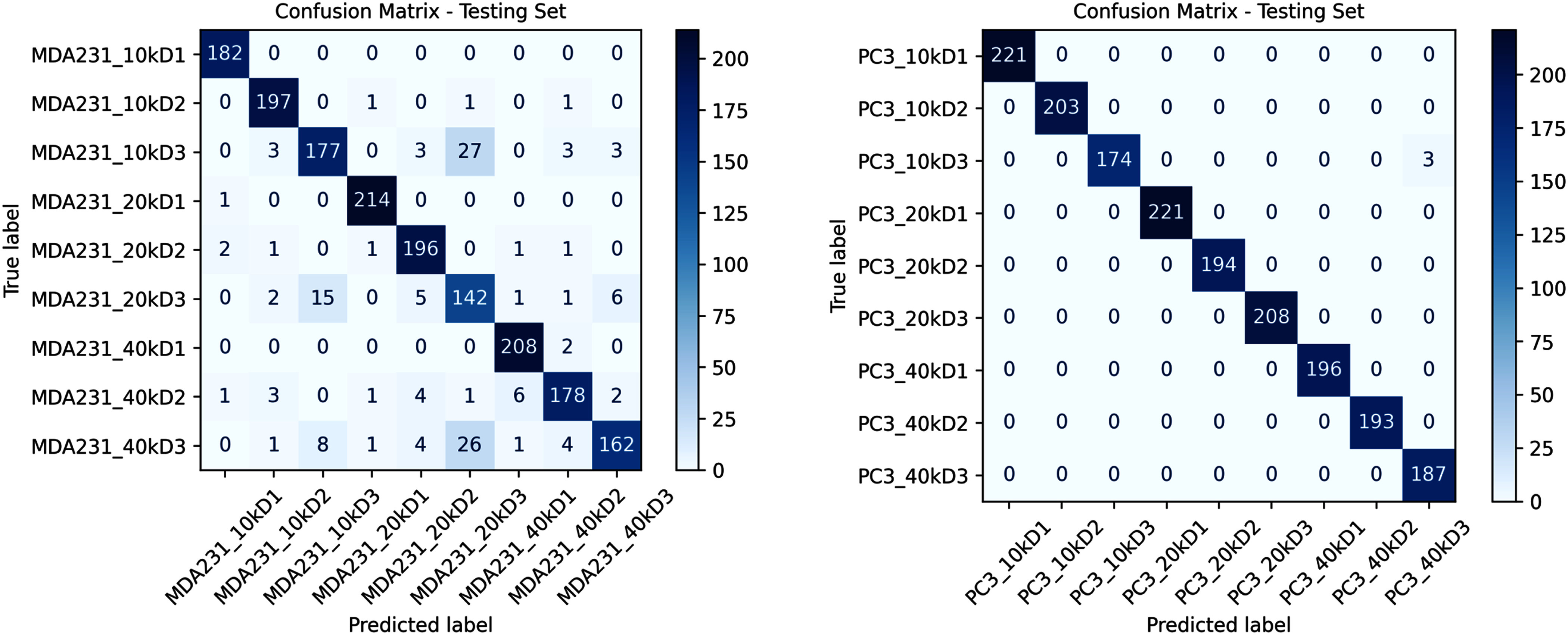
Confusion matrix showing classification performance of the model across 9 classes of the MDA-MB-231 and PC3 dataset.

The trained models were then tested on the test set. The confusion matrices of the tests, Fig. 6, demonstrate that ViTs achieved very high performance on both datasets, with fewer misclassifications on the PC3 dataset. This aligns with the model’s training behavior, where PC3 showed faster convergence and more stable performance in general.

The ViT method achieved an overall F-1 accuracy of 0.88 for the classification of MDA-MB-231 images, with both the macro and weighted averages of accuracy at 0.88. Some classes, such as those with “10 k” density and earlier day points (D1, D2), show high precision and recall, while the others demonstrate lower scores. In comparison, the ViT achieved an F-1 accuracy of 0.98 in PC3 dataset, with macro and weighted averages both at 0.99. This comparison suggests that MDA-MB-231 dataset presents greater classification challenges than PC3.

**Fig. 7.**
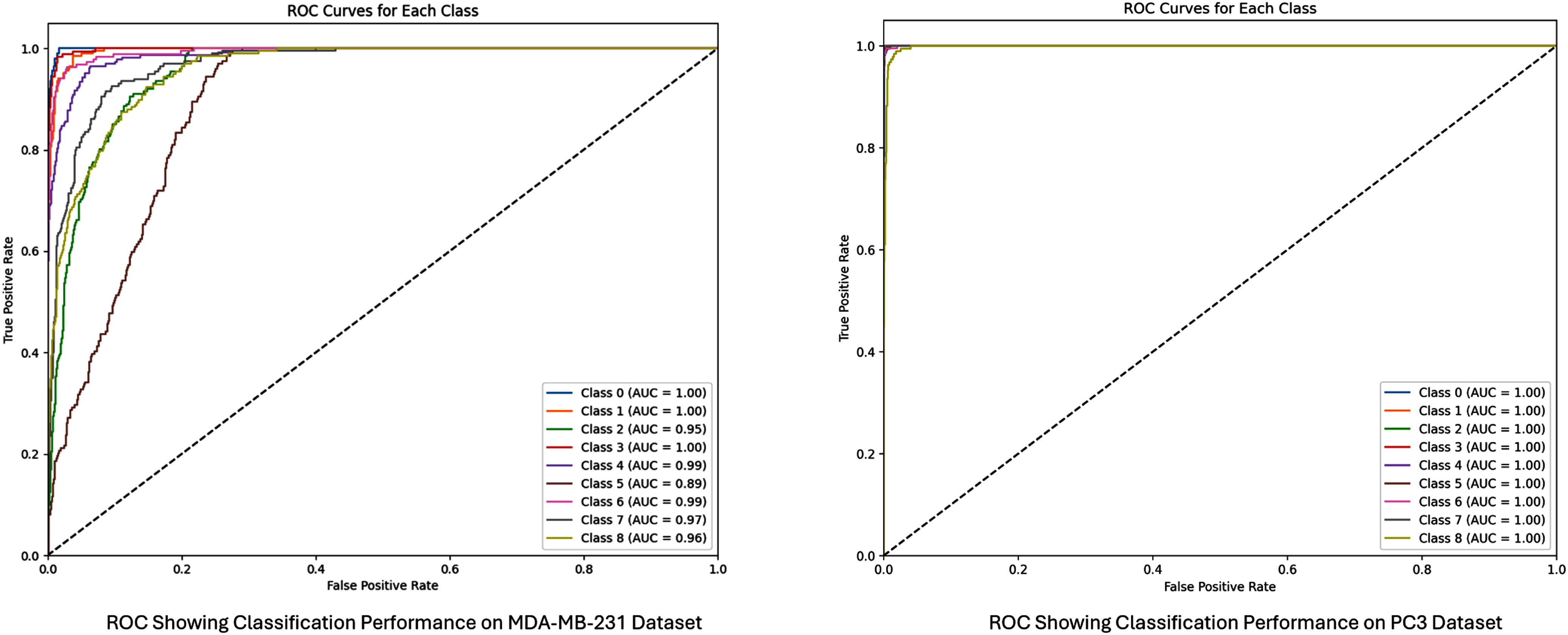
ROC showing classification performance of the ViT model across 9 classes of the MDA-MB-231 and PC3 dataset.

Fig. 7 shows the ROC curves on the test set for the two cell lines, respectively. The ROC curves for the 9 classes of the PC3 dataset clustered tightly. They all rise steeply toward the top-left corner and then remain flat, indicating extremely high true-positive rates at very low false positives; this is further confirmed by an micro-average AUC of 0.99. In comparison, the ROC curves for MDA-MB-231 dataset spread more widely. They all rise in a less steep ascent toward the top-left corner. The micro-average AUC for MDA-MB-231 is 0.94, which still indicates good classification but not as good as that for PC3. The spread between different ROC curves in MDA-MB-231 is also indicative of the variation in classification difficulty across different classes; in contrast, PC3’s tightly clustered, nearly overlapping curves suggest consistent performance across all classes.

## IV. Discussion

*Interpretability and Visualization* The proposed Vision Transformer (ViT) provides a comprehensive analysis of the model’s decision behavior through attention heatmaps and gradient-based saliency maps shown in Fig. 4. The attention maps highlight the spatial focus of transformer heads, demonstrating that the model primarily attends to morphologically significant regions such as cell membranes, nuclei, and cytoplasmic textures, while the saliency maps capture pixel-level importance contributing to classification outcomes. Together, these visualizations confirm that the ViT effectively captures biologically meaningful features and enables transparent, interpretable decision-making. Furthermore, as summarized in Table III, the ViT outperforms CNN-based architectures, while attention-enhanced variants (CNN-SE and CNN-CBAM) substantially close the performance gap by improving long-range feature modeling and relevance weighting. This combined analysis underscores both the superior accuracy and the biological interpretability of the proposed ViT framework.

## V. Conclusion

*Summary of Findings:* A consistent preprocessing pipeline (Otsu thresholding, morphological filtering, watershed) and a 5-fold cross-validation grid search ensured fair comparisons and robust estimates. The results indicate that (i) attention narrows the gap between CNNs and transformers while retaining CNN efficiency, and (ii) transformers remain the best end-to-end choice when dataset complexity demands global context.

These findings support practical deployment paths: attention-augmented CNNs offer efficient, high-quality baselines for routine analysis, whereas ViTs provide peak performance where accuracy is paramount. Future work will extend to temporal sequences and additional cell lines, explore domain adaptation and self-supervised pre-training, and examine calibration and interpretability to facilitate reliable clinical use.

*Limitations:* Evaluation is restricted to two cell lines under a single phase contrast microscopy setting and is based on 2D single frame models.

*Future work:* Broaden datasets and acquisition conditions, incorporate temporal (sequence) modeling, and conduct systematic robustness studies to noise, blur, and illumination variation.

## Supplementary Materials

The experimental data and implementations used in this study are publicly available at https://github.com/noreenfayyaz/Vision-Transformer-for-High-Precision-Classification-of-Cancer-Cell-Cultures.
